# Elevated neoantigen levels in tumors with somatic mutations in the HLA-A, HLA-B, HLA-C and B2M genes

**DOI:** 10.1186/s12920-019-0544-1

**Published:** 2019-07-25

**Authors:** Andrea Castro, Kivilcim Ozturk, Rachel Marty Pyke, Su Xian, Maurizio Zanetti, Hannah Carter

**Affiliations:** 1 Department of Medicine, Division of Medical Genetics, University of California San Diego, La Jolla, CA 92093 USA; 2 Bioinformatics and Systems Biology Program, University of California San Diego, La Jolla, CA 92093 USA; 3 Moores Cancer Center, University of California San Diego, La Jolla, CA 92093 USA; 4 The Laboratory of Immunology and Department of Medicine, University of California San Diego, La Jolla, CA 92093 USA; 5 CIFAR, MaRS Centre, West Tower, 661 University Ave., Suite 505, Toronto, ON M5G 1M1 Canada

## Abstract

**Background:**

The major histocompatibility complex class I (MHC-I) molecule is a protein complex that displays intracellular peptides to T cells, allowing the immune system to recognize and destroy infected or cancerous cells. MHC-I is composed of a highly polymorphic HLA-encoded alpha chain that binds the peptide and a Beta-2-microglobulin (B2M) protein that acts as a stabilizing scaffold. HLA mutations have been implicated as a mechanism of immune evasion during tumorigenesis, and B2M is considered a tumor suppressor gene. However, the implications of somatic HLA and B2M mutations have not been fully explored in the context of antigen presentation via the MHC-I molecule during tumor development. To understand the effect that B2M and HLA MHC-I molecule mutations have on mutagenesis, we analyzed the accumulation of mutations in patients from The Cancer Genome Atlas according to their MHC-I molecule mutation status.

**Results:**

Somatic B2M and HLA mutations in microsatellite stable tumors were associated with higher overall mutation burden and a larger fraction of HLA-binding neoantigens when compared to B2M and HLA wild type tumors. B2M and HLA mutations were highly enriched in patients with microsatellite instability. B2M mutations tended to occur relatively early during patients’ respective tumor development, whereas HLA mutations were either early or late events. In addition, B2M and HLA mutated patients had higher levels of immune infiltration by natural killer and CD8+ T cells and higher levels of cytotoxicity.

**Conclusions:**

Our findings add to a growing body of evidence that somatic B2M and HLA mutations are a mechanism of immune evasion by demonstrating that such mutations are associated with a higher load of neoantigens that should be presented via MHC-I.

**Electronic supplementary material:**

The online version of this article (10.1186/s12920-019-0544-1) contains supplementary material, which is available to authorized users.

## Introduction

Immune evasion is one of the hallmark traits characteristic of cancer cells [1]. The near universal requirement for tumor cells to evade immune elimination implicates the immune system as a major selective force acting on developing tumors. When a tumor cell successfully evades the immune system, the mutations harbored within can persist and propagate as the cell divides.

In humans, the *HLA-A*, *HLA-B*, and *HLA-C* genes encode major histocompatibility complex class I (MHC-I) molecules, which display intracellular peptides on the cell surface for inspection by CD8+ T cells. These T cells have the potential to recognize the MHC-I-peptide complex and become activated cytotoxic T cells (CTLs). Cancer immunotherapies that target CTL activation rely on clinical selection of appropriate neoantigens, mutated peptides specific to tumor cells, to stimulate a response [2]. Although these cancer immunotherapies are of high interest to patients and clinicians, they have not yet shown widespread clinical success [3].

The MHC-I molecule is composed of a highly polymorphic HLA encoded alpha chain and a beta-2-microglobulin (B2M) protein that acts as a stabilizing scaffold. B2M is essential for MHC-I complex formation and peptide presentation. B2M mutations and loss of heterozygosity (LOH) are linked to decreased MHC class I expression and decreased patient survival [4, 5]. In addition to HLA-A, HLA-B, and HLA-C, B2M binds to other immune proteins including CD1, FCGRT, HFE, HLA-E, HLA-G LILRB, and MR1. Somatic mutations in HLA-A and HLA-B have also been shown to be under positive selection during tumorigenesis and are more frequent when tumor immune cell infiltration and cytotoxicity are high [6]. Importantly, MHC-I molecule presence on the cell surface can provide an inhibitory signal to natural killer (NK) cell mediated effector functions [7]. In addition to classical HLA molecules HLA-A, HLA-B, HLA-C, and HLA-G, nonclassical HLA-E acts as a ligand to inhibitory receptors on NK cells. Thus, both presence and antigen presentation function of MHC-I molecules contribute to anti-tumor immunity.

A recent study found that an individual’s HLA genotype can facilitate immune evasion and shape the landscape of a patient’s acquired mutations [8]. Somatic mutations generating peptides with low affinity for an individual’s respective HLA alleles were likely to evade immune detection and persist in the tumor. Somatic LOH in the human leukocyte antigen (HLA) locus is thought to impair immune surveillance and was reported to occur in 40% of non-small-cell lung cancers. The authors found that HLA LOH was significantly associated with a high mutational burden and cancer-specific neoantigens generated from these mutations were biased to bind to the lost HLA allele [9]. Thus, immune evasion may depend on an individual’s unique HLA genotype and the specificity of neoantigens for particular HLA alleles.

**Fig. 1:**
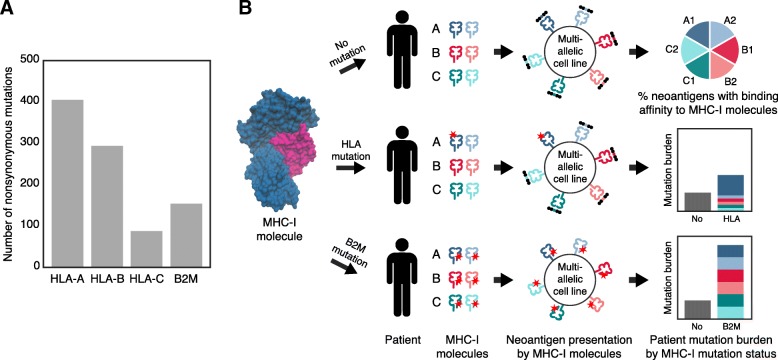
Somatic mutations affecting components of the MHC-I molecule. **a** The total number of nonsynonymous mutations targeting the genes encoding the components of the MHC-I complex, B2M and HLA-A, HLA-B or HLA-C proteins, across all TCGA patients. The HLA mutation counts were obtained via Polysolver. **b** Schematic representation of the effects of mutations that alter the cell surface composition of MHC-I. An HLA mutation will affect a specific MHC-I molecule, whereas a B2M mutation will affect all MHC-I molecules; both mutations can increase the mutation burden of the patient. In the case of an HLA mutation, the patient mutation burden should include more neoantigens with affinity for the mutated HLA allele. The MHC-I molecule displayed is composed of B2M (pink) and HLA-A (blue) proteins (PDB: 3bo8)

We previously observed an uncharacteristic enrichment of somatic mutations at B2M interaction partner binding interfaces [10]. Whereas for most cancer genes, mutations occurred preferentially on the cancer gene itself, genes encoding B2M binding partners showed almost as many somatic mutations as B2M (Fig. 1a). Given B2M’s role as a central component of MHC-I, we hypothesized that mutations affecting B2M’s interaction with HLA-A, HLA-B, and HLA-C could facilitate immune evasion by altering the availability of MHC-I molecules with distinct specificities, thus affecting presentation of specific peptides to the immune system (Fig. 1b). To gain a better understanding of the role of somatic mutations affecting B2M and its partners in immune evasion, we examined their effect on mutation burden, antigen binding affinity, immune infiltration, and cytotoxicity in tumors sequenced by The Cancer Genome Atlas (TCGA).

## Results

### HLA and B2M mutations in TCGA

**Fig. 2:**
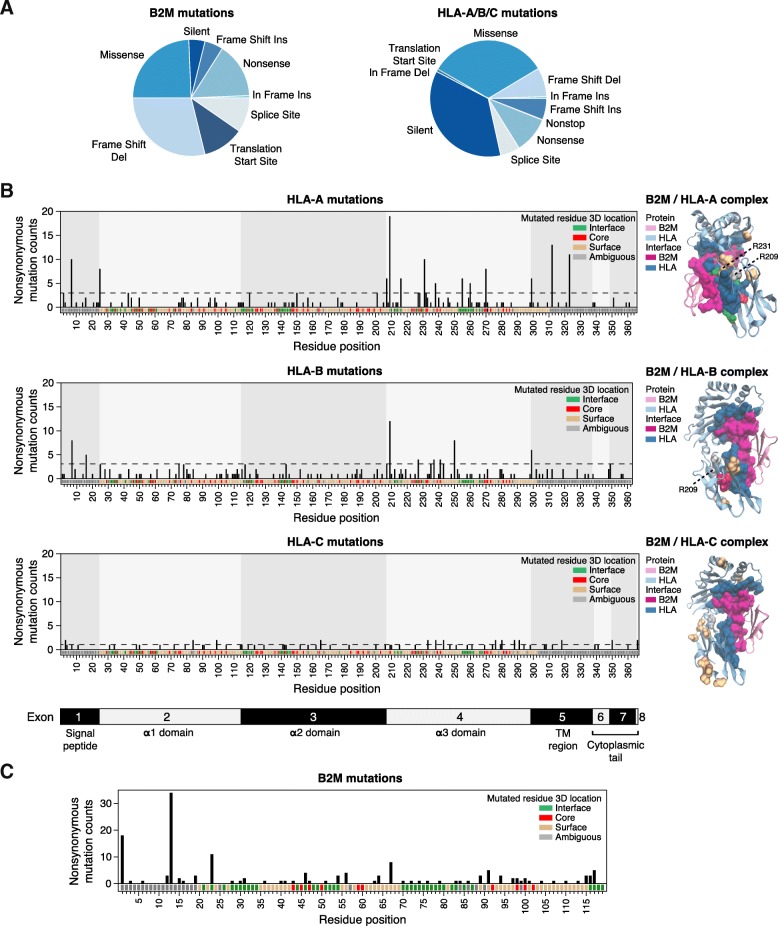
Mutational analysis of MHC-I complex. **a** Pie charts displaying percentages of types of mutation for the B2M protein; and for the combined HLA-A, HLA-B and HLA-C proteins, respectively, across all TCGA patients. **b** Distribution of nonsynonymous mutation counts, obtained from Polysolver, for HLA-A, HLA-B, and HLA-C proteins, across functional domains. The corresponding functional domains of HLA proteins are shown at the bottom. The UniProt sequential residue numbering scheme is used for residue numbering, which requires subtraction of the signal peptide (24 residues) for mapping to the IMGT/HLA residue numbering scheme. On the right, 3D crystal structures of MHC-I complex are displayed as B2M and HLA-A complex (PDB: 3bo8), as B2M and HLA-B complex (PDB: 3b3i), and as B2M and HLA-C complex (PDB: 4 nt6). Purple ribbons indicate B2M protein, while blue ribbons indicate the HLA proteins. The highlighted purple and blue residues correspond to the interface regions of B2M and HLA proteins, respectively. Hotspot mutations for HLA proteins (frequency > 3 for HLA-A, frequency > 3 for HLA-B, and frequency > 1 for HLA-C) are highlighted as green, red, tan and gray indicating interface, core, surface, and ambiguous residues, respectively. **c** Distribution of nonsynonymous mutation counts across the entire B2M protein. On the bottom of the plot, all amino acid residues of B2M protein are colored based on their 3D location: interface, core, surface, or ambiguous

B2M mutation calls were obtained directly from the MAF files provided by TCGA. Because the HLA locus is highly polymorphic, mutation calls against the reference genome are unreliable. Instead, we ran Polysolver [6] to simultaneously call patient-specific HLA types and detect somatic mutations affecting a patient’s HLA alleles. Out of 10,428 TCGA patients that had the necessary whole exome sequencing data, only 579 patients had an HLA mutation and 125 patients had B2M mutations. Most of these mutations were nonsynonymous (Fig. 2a). To determine whether nonsynonymous mutations occurred at amino acid residues with the potential to interfere with formation of the MHC-I molecule, experimental 3D structures for the B2M-HLA complex were obtained from the Protein Data Bank [11] and used to annotate amino acid residue location at protein core, surface or at the physical interface between B2M and HLA encoded proteins (Methods).

Mutations on HLA proteins, particularly HLA-A, showed a biased distribution with several recurrent hotspots (Fig. 2b). Mutations were most concentrated in the α3 domain that mediates interaction with the T cell receptor (TCR) (206 mutations, 40.63% of total; OR = 2.04, *p* < 2.58e-09), and included multiple recurrent hotspots. Fifty-one mutations (10.06%) were observed in the transmembrane domain including additional hotspots. Although mutations were observed throughout the α1 and α2 domains that form the peptide binding groove, they tended to be less recurrent (88 mutations, 17.36% for both α1 and α2). This may reflect the much larger heterogeneity of this region across HLA alleles.

Recurrent hotspot mutations often targeted interface and core regions on HLA-A, while they targeted core and surface regions on HLA-B, and surface regions on HLA-C (Fig. 2b). Since there are many alleles for each HLA protein, we used the consensus of residue annotations across different alleles to annotate each HLA protein (Additional file 1: Figure S1). Even though the annotations for most frequently mutated residues were in agreement between different HLA alleles, there were some exceptions, including residue 231 on HLA-A. Although residue 231 (R231) on HLA-A was annotated as surface based on the consensus across HLA-A alleles, the residue is located very close to the interface region (Fig. 2b) and in fact was predicted as an interface residue on 2 of the 6 HLA-A allele structures analyzed. Additionally, although residue 209 (R209) on HLA-A and HLA-B proteins was annotated as ambiguous due to its intermediate value of relative solvent accessible surface area (RSA) for most HLA-A/B structures analyzed, the average RSA across structures is close to the threshold for core annotation (7.17), and R209 was indeed annotated as core in some of them. Overall, the distribution of HLA mutations for the three proteins was consistent with the previous report by Shukla et al. [6], though the current analysis incorporates an overall larger number of samples. Mutations in B2M were largely loss of function (Fig. 2a) and more broadly distributed (Fig. 2c), as expected for a tumor suppressor gene, though several positions were also recurrently mutated.

### Expected effects of B2M versus HLA mutations on MHC-I composition

Since B2M is an essential component of all MHC-I molecules, loss of B2M should equally impact MHC-I molecules derived from different HLA alleles. The B2M interface with HLA alleles is shared across the different alleles (Additional file 2: Figure S2), so mutations at this interface are also likely to affect all variants of an individual’s MHC-I molecule, although complexes involving B2M and binding partners that use alternative interfaces should not be affected. In contrast, loss of function or interface mutations affecting a specific HLA allele would only affect the MHC-I molecules derived from that allele. Thus, we speculate that B2M mutations are likely to reduce the total amount of MHC-I molecules presenting antigens on the tumor cell surface, while HLA mutations would impact which mutations could be presented as neoantigens.

### Mutations in MHC-I proteins are associated with increased mutation burden

We hypothesized that both B2M and HLA mutations would affect MHC-I presentation of mutations. Mice with total lack of B2M express little if any cell surface MHC-I and lack cytotoxic CD8+ T Cells [12, 13]. In human lung cancers, an association was found between higher somatic mutation burden and HLA loss of heterozygosity [9]. If somatic mutations to HLA and B2M similarly impair antigen presentation, we would expect to see an increased mutation burden when comparing to unmutated patients.

**Fig. 3:**
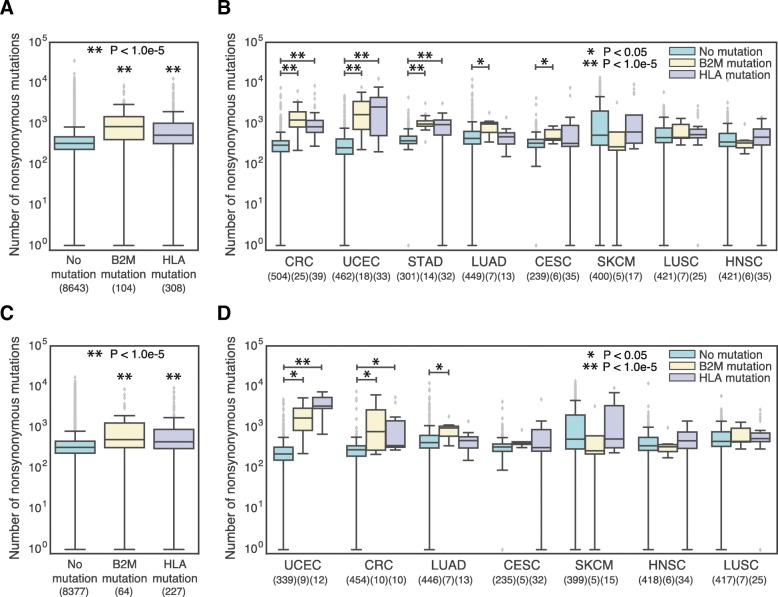
Increased mutational burden is related to mutations in MHC-I. **a** and **c** Boxplots showing the total number of expressed nonsynonymous mutations of TCGA patients who acquired a mutation in their B2M protein or in one of their HLA alleles versus patients who did not acquire any B2M or HLA mutation, (**a**) for all patients, and (**c**) for only MSS patients. Sample sizes for each patient group are written under their name. **b** and **d** Boxplots showing total number of expressed nonsynonymous mutations for TCGA patients with or without B2M or HLA mutations, (**b**) for all patients, and (**d**) for only MSS patients. Patients are divided by tumor type and only the tumor types with at least 5 mutated patients are shown. *P*-values are adjusted for multiple comparisons using the Benjamini–Hochberg procedure. Sample sizes for each patient group are written under the tissue name

We first analyzed 9055 TCGA patients across 31 solid tumor types that had both exome and RNA sequencing data (Fig. 3a), removing patients that had synonymous B2M or HLA mutations. We then performed a cancer-specific analysis of 3514 patients across 8 solid tumor types with at least 5 somatic B2M and HLA mutations (Fig. 3b, Additional file 3: Figure S3A). To determine whether somatic mutations to B2M and HLA were associated with an overall higher mutation burden, we compared the total number of expressed nonsynonymous mutations in patients with and without nonsynonymous somatic B2M or HLA mutations. Overall, we observed that both patients with a B2M and an HLA mutation had significantly higher tumor mutation burdens (Mann Whitney test, B2M *p* < 1.1e-20 and HLA *p* < 1.1e-30) than patients without (Fig. 3a). Pan-cancer, B2M mutated patients also had significantly higher mutation burdens than HLA mutated patients (Mann Whitney test, *p* < 0.0028). There were approximately equal numbers of early stage (I & II) and late stage (III & IV) tumors in these three groups (Additional file 4: Figure S4). We repeated the pan-cancer mutational burden analysis with Cancer Cell Line Encyclopedia (CCLE) data for 25 B2M-mutated cell lines, 114 HLA cell lines, and 1381 non-mutated cell lines, and observed the same trend: cell lines with B2M and HLA mutations had significantly higher overall mutational burden than cell lines without (Additional file 5: Figure S5). When we analyzed tumors by tissue type, we observed that certain cancers (stomach adenocarcinoma, endometrial cancer, colorectal cancer, lung adenocarcinoma, and cervical cancer) also had significantly higher mutational burden in mutated patients (Fig. 3b). Stomach, uterine and colorectal cancers have documented high rates of microsatellite instability (MSI), thus we evaluated whether B2M and HLA mutations were biased to occur in high MSI tumors. Using MSI annotations available for 10,415 patients from Kautto et al. [14], we found a significant bias for B2M and HLA mutations to occur in patients with MSI (Fisher’s exact test; B2M OR = 14.66, *p* < 8.7e-24; HLA OR = 6.28, *p* < 2.0e-36). To rule out the possibility that MSI was solely driving our results, we reanalyzed the mutational burden between B2M and HLA mutated and unmutated patients, this time retaining only 8668 microsatellite stable (MSS) patients. Interestingly, we found similar trends in elevated mutational burden associated with B2M and HLA mutation (Fig. 3c, d, Additional file 3: Figure S3B), and consequently focused on MSS patients only in the subsequent analyses. Thus, even in MSS tumors, B2M and HLA mutations are associated with an increased nonsynonymous mutational burden.

### Mutations in MHC-I proteins are associated with increased binding neoantigen counts

To obtain more evidence as to whether the elevated mutation counts observed in HLA and B2M mutated patients were a result of the mutation, or vice versa, we compared the fraction of mutations likely to generate neoantigens across MSS patients with and without B2M and HLA mutations. We speculated that if B2M and HLA mutations are an artifact of higher mutation rates, the proportion of mutations that generate neoantigens should not differ relative to patients without such mutations. However, if these mutations truly facilitate immune escape, neoantigens should be enriched among the observed mutations.

**Fig. 4:**
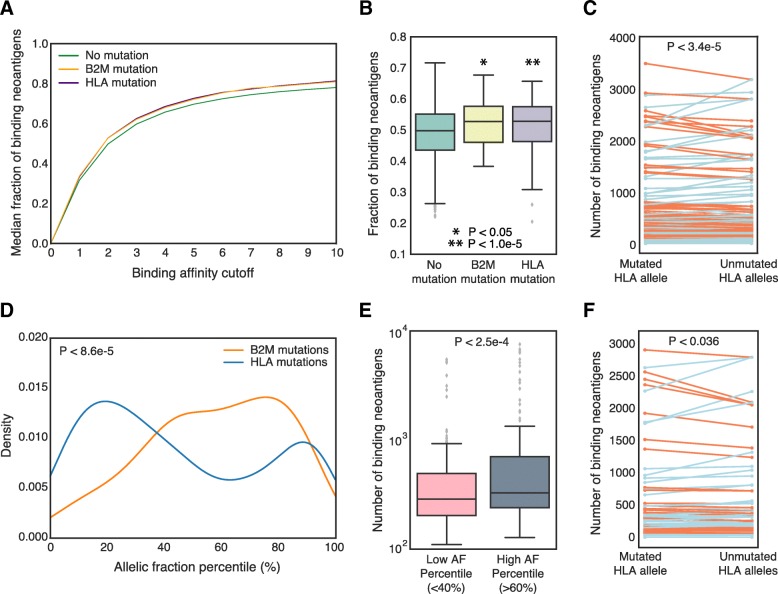
Analysis of binding neoantigens to patient HLA alleles. **a** Empirical cumulative distribution function showing the proportion of expressed missense and indel mutations labeled as binding neoantigens at different PHBR-I score cutoffs in MSS patients. **b** Boxplots comparing the fraction of binding neoantigens in tumors with no B2M or HLA mutation (teal) versus patients with a B2M mutation (yellow) or HLA mutation (purple). A PHBR-I score cutoff of 2 was used to designate a binding neoantigen for this comparison. **c** Total number of neoantigens that bind to a patient’s mutated HLA allele versus the average number of neoantigens across the unmutated HLA alleles across all cancer types for MSS patients. A red line indicates that there are more neoantigens with binding affinity to the mutated HLA allele than the average across the unmutated HLA alleles; and a blue line depicts the opposite trend. **d** Allelic fraction percentile distribution for expressed mutations in MSS patients with B2M and HLA mutations. We used the Kolmogorov-Smirnov statistic to determine whether the two distributions were significantly different. **e** Comparison of the number of expressed neoantigens with binding affinity to the patient-specific mutated allele between the low AF percentile (< 40%) and the high AF percentile (> 60%) HLA mutated patients. Patients with MSI and with mutations in both B2M and HLA genes were excluded. **f** Comparison of the total number of neoantigens that bind to a patient-specific mutated HLA allele versus the average number of neoantigens with binding affinity to the five unmutated HLA alleles in patients with high allelic fraction percentile HLA mutations

Using HLA allele genotypes called by Polysolver [6], we calculated patient-specific MHC-I presentation scores for all expressed mutations observed in each patient’s tumor [8, 15]. We previously demonstrated that these affinity-based presentation scores, called PHBR-I scores, can distinguish peptides found in complex with MHC-I on the cell surface in mass spectrometry experiments from random peptides simulated from the human proteome, supporting that affinity is a reasonable proxy for cell surface presentation [8]. Indeed, when we looked at the fraction of expressed mutations considered to be neoantigens at various PHBR-I cutoffs, we found that at any given cutoff, a higher fraction of mutations represented neoantigens in both B2M and HLA mutated patients (Figs. 4a, b). This corresponded to overall higher numbers of neoantigens in B2M and HLA mutant tumors (Additional file 6: Figure S6). The higher overall proportion of neoantigens is consistent with both somatic B2M and HLA mutations impairing presentation of neoantigens for immune surveillance.

### Assessing bias in neoantigen affinities in patients with mutant HLA alleles

McGranahan et al. reported that in lung cancer, subclones that had lost a particular HLA allele tended to accumulate mutations with higher affinity for the lost allele, suggesting that such mutations were no longer subject to immunoediting [9]. We therefore sought to assess whether mutations accumulating in tumors with HLA mutations showed a bias in affinity toward the affected HLA allele. We first evaluated whether the number of mutant-allele specific mutations in these patients was higher than the average number of mutations specific to each of the other alleles (Fig. 4c). We observed several patients for which the number of mutant-allele specific mutations was indeed higher (Fig. 4c; red lines). We note that the current study design differs from the study by McGranahan et al. in that we do not have subclone-specific sequencing data, and thus can not determine which mutations occurred in the same cell population as the mutated HLA allele. We also did not consider allele-specific deletion events, and thus the assumption that the other 5 HLA alleles are intact may be incorrect for some patients.

### Timing of somatic mutations in MHC-I proteins

To better understand B2M and HLA mutation timelines, we analyzed the tumor allelic fraction of expressed mutations for all patients. Early clonal mutations are present in a larger fraction of cancer cells than later subclonal mutations and are, therefore, expected to be present in a higher fraction of the reads generated from that site during tumor sequencing. Although this assumption can be complicated by sampling bias and genomic instability of tumors, we nonetheless expect that somatic point mutations with higher read support will in general have occurred at earlier time points than those with lower read support. Since each individual’s tumor is unique, we quantified B2M and HLA mutations in terms of their allelic fraction percentile relative to other mutations observed in the same tumor (Fig. 4d). Interestingly, B2M mutations tended to be present at higher percentiles than most HLA mutations, suggesting that B2M mutations might occur earlier in tumor development and affect a higher proportion of tumor cells. Most HLA mutations had low percentiles, suggesting these were late, subclonal events, while a subset had high percentiles and likely occurred early during tumor development in those individuals. This observation agrees with the previous report by McGranahan et al. that found HLA loss in lung cancer to be predominantly subclonal with a few observations of clonal loss noted. Patients with MSI tended to have HLA mutations with higher variant allele fraction (VAF) (Fisher’s exact test, OR = 73.3, *p* < 8.1e-16). These findings remained even when we considered only mutations in regions unaffected by copy number changes which can confound VAF estimates (Additional file 7: Figure S7). Interestingly, we found that tumors with early HLA mutations had significantly higher levels of neoantigens predicted to specifically bind to the mutated allele than tumors with late HLA mutations (Fig. 4e). When we evaluated the bias in specificity of neoantigens for the mutated allele in patients with early HLA loss, we found a significant difference in the number of binding neoantigens between the mutated HLA allele and average of unmutated HLA alleles (Fig. 4f). We conclude that somatic B2M and HLA mutations are associated with an overall higher burden of neoantigens, supporting the notion that these mutations facilitate tumor immune escape.

### Correlation of B2M versus HLA mutation with immune cell infiltration and cytotoxicity

Effective antigen presentation via MHC-I is associated with CD8+ T cell driven cytotoxicity. Furthermore, cell surface MHC-I molecules deliver an inhibitory signal to natural killer (NK) cells. Thus, changes to cell surface presentation of neoantigens by MHC-I due to mutations in B2M and HLA may be reflected in immune cell infiltration levels and levels of cytotoxicity. We quantified immune cell infiltration from tumor RNA sequencing data using Cibersort [16] and levels of cytotoxicity using the score proposed by Rooney et al. [17]. While Shukla et al. previously evaluated immune infiltrates and cytotoxicity in the context of somatic HLA mutations, to our knowledge B2M mutations have not previously been analyzed in this context [6].

**Fig. 5:**
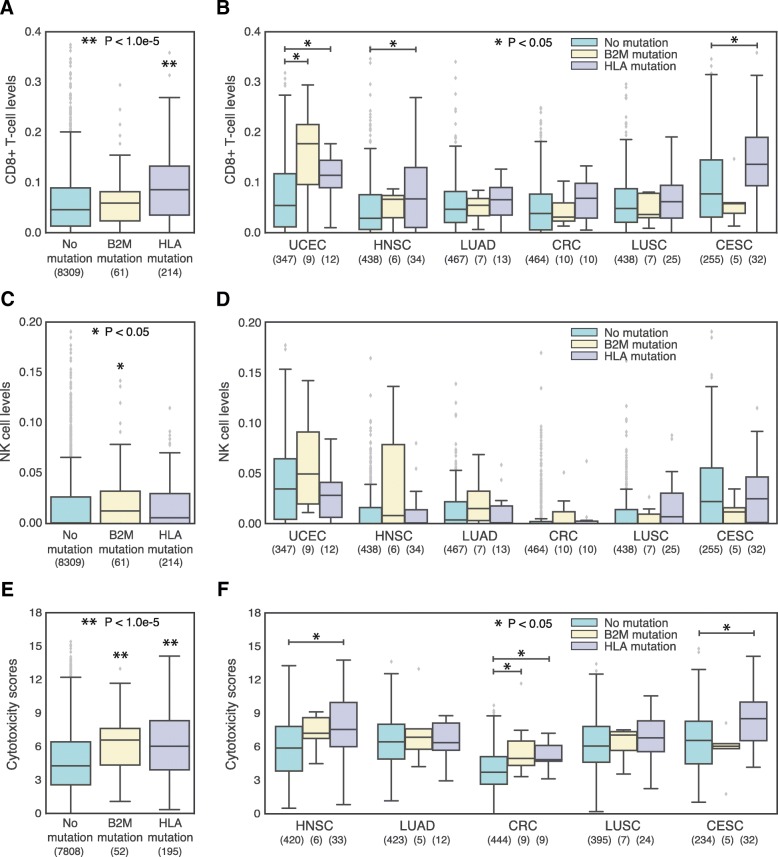
Increased NK, CD8+ T-cell and cytotoxicity levels are associated with mutations in MHC-I. **a** and **c** and **e** Boxplots comparing MSS TCGA patients with or without B2M or HLA mutations, in terms of their (**a**) CD8+ T cell levels, (**c**) natural killer (NK) cell levels, and (**e**) cytotoxicity scores. Sample sizes for each patient group are written under their name. **b** and **d** and **f**) Boxplots comparing MSS TCGA patients with or without B2M or HLA mutations, in terms of their (**b**) CD8+ T cell levels, (**d**) natural killer (NK) cell levels, and (**f**) cytotoxicity scores. Patients are divided by tumor type and only the tumor types with at least 5 mutated patients are shown. *P*-values are adjusted for multiple comparisons. Sample sizes for each patient group are written under the tissue name

CD8+ T cell levels were elevated in tumors with HLA mutations, both pan-cancer (Fig. 5a) and in several tumor types (Fig. 5b, Additional file 8: Figure S8A). A possible explanation is that CD8+ T cells are primed in secondary lymphoid organs and travel to the tumor where they accumulate due to the lack of the corresponding MHC-I molecule / peptide complex. NK cell levels were elevated in tumors with B2M mutations pan-cancer (Fig. 5c), however the levels were not significantly different in any given tumor type (Fig. 5d, Additional file 8: Figure S8B). Loss of B2M resulting in reduced cell surface MHC-I molecules should reduce the ability of tumor cells to inhibit NK cell driven cytotoxicity, however it is unclear whether this would affect NK cell levels in the tumor. Cytotoxicity was elevated in both HLA and B2M mutant tumors pan-cancer (Fig. 5e) and in several tumor types (Fig. 5f, Additional file 8: Figure S8C). These trends are consistent with the idea that mutations are a mechanism of escape from immune surveillance, as previously suggested by Shukla et al. for HLA mutations [6].

## Discussion

Many immunotherapies, such as immune checkpoint inhibitors, rely on the integrity of patient’s immune system to eliminate tumors. Tumors use a variety of strategies to evade the immune system, raising important questions about how different mechanisms of immune evasion could impact response to particular immunotherapies. We found that somatic point mutations in proteins comprising the MHC-I, B2M and HLA, showed signs of positive selection in tumors. This observation motivated our study of the effects of somatic B2M and HLA mutations on accumulation of putative neoantigens in tumors.

Our analysis builds on work by Shukla et al. that first applied Polysolver to evaluate patterns of HLA mutation across tumors and showed that such mutations occurred preferentially in tumors with high mutation burden and under strong pressure by the immune system as evidenced by high levels of CD8+ T cell infiltration [6]. Here we further analyze patterns of mutation in tumors with HLA mutations, incorporating information about which mutations are likely to be presented by MHC-I molecules derived from patient-specific HLA alleles, and comparing to tumors with B2M mutations or with unaltered MHC-I. Our analysis supports a model where B2M mutations reduce the overall levels of cell surface MHC-I molecules while HLA mutations perturb the overall composition of the MHC-I complex landscape, both providing escape from immune surveillance. Our findings are consistent with those of McGranahan et al. who reported that somatic loss of heterozygosity in the *HLA* locus was a common mechanism of immune evasion, and that loss of a specific HLA allele could render a subset of neoantigens within the tumor ineffective at generating an immune response upon checkpoint inhibition [9]. While both B2M and HLA mutated patients showed elevated mutation rates, we observed differences in how neoantigens accumulated in these tumors, with B2M mutant tumors harboring the most neoantigens and tumors with intact MHC-I molecules harboring the least.

Notably, B2M mutations were highly enriched in tumors with microsatellite instability, a phenomenon that has been previously observed in the context of colorectal cancer [18] and is now confirmed for other tumor types with high MSI. MSI tumors were associated with higher immune cell infiltration and robust immune responses in this disease [19]. Previous studies have also linked B2M mutations to increased levels of local immune cytolytic activity in uterine, stomach, colorectal and breast cancer [17]. It remains unclear to what extent high mutation burden precedes immune infiltration, cytotoxicity and escape via B2M or HLA mutation, or whether the rate and affinity characteristics of the mutations that occur after the event differ from those before. Grasso et al. [20] showed that MSI-H colorectal tumors disrupt B2M and HLA genes independent of mutational load with direct effect on T cell infiltration. We conclude that mutations to either component of MHC-I will provide effective escape in the setting of a robust anti-tumor immune response, however mutations to B2M may be more beneficial in settings such as MSI when the number of neoantigens generated is highest.

We note that the current analysis has several limitations. First, our analysis only considered mutations in HLA alleles, whereas other types of variation, including loss of heterozygosity or lack of expression could confer similar effects. In the current analysis, patients with such effects would be grouped with non-mutated tumors, which would reduce the statistical power of the analyses that we performed. In addition, we did not have information about the subclonal membership of particular mutations within the tumor, and thus could not distinguish mutations occurring in the subset of tumor cells with HLA mutation from other mutations in the tumor. Knowledge of the subclonal architecture of the tumor would be helpful to fully investigate the affinity bias of new mutations for the mutated HLA allele. Future studies should address these shortcomings.

## Conclusion

Here we show that somatic mutations affecting B2M and HLA genes interact with the accumulation of somatic mutations that generate neoantigens during tumor development. Mutations in both genes relieve pressure by the immune system, allowing the tumor to evade an active immune response. A better understanding of how these mutations differ in shaping the oncogenic landscape may provide insights as to how these factors could contribute to resistance to therapies that induce strong local anti-tumor immunity.

## Methods

### Data

All available whole exome sequencing (WXS) data as of 5/3/2018 was downloaded from The Cancer Genome Atlas (TCGA) database via their Genomic Data Commons (GDC) client. Both .bam files and auxiliary .bai files were downloaded. All available somatic mutation data as of 5/21/2018 was downloaded from the TCGA database in the form of TCGA project mutation annotation files (MAF). Clinical data were also obtained from the GDC (downloaded on 4/25/2017) (Additional file 9: Table S1).

### Protein structure analysis

Experimental 3D X-ray protein structures for the B2M and HLA-A/B/C complexes were obtained from the Protein Data Bank (PDB) [11]. Amino acid residues of each PDB structure were annotated based on their 3D location in the protein as core and surface according to their relative solvent accessible surface area (RSA) calculated using Naccess [21]. Residues with RSA higher than 15 were annotated as surface and residues with RSA lower than 5 were annotated as core, while residues with RSA values between 5 and 15 were annotated as ambiguous. Residues involved in the physical interaction between B2M and HLA proteins are predicted using KFC2 [22] and annotated as interface. PDB residue positions were mapped onto the UniProt residue positions using the PDBSWS server [23]. UniProt residues are numbered based on their position in the protein sequence of the full-length protein, starting from 1. If multiple PDB structures were available for the same protein, we took consensus as the final annotation; and in the case of a tie, the residue was labeled as ambiguous. The residues without known 3D structure are also labeled as ambiguous. We had structures for 6 alleles of HLA-A protein, 15 alleles of HLA-B protein, and 3 alleles of HLA-C protein. We took consensus of residue annotations of different HLA alleles to annotate each HLA protein. VMD [24] is used to visualize protein 3D structures (Fig. 2b). Exon information for HLA proteins is obtained from the IMGT/HLA database (v3.34; https://www.ebi.ac.uk/ipd/imgt/hla/) [25].

### HLA typing and mutation calling

HLA genotyping and mutation calling was performed for *HLA-A*, *HLA-B*, and *HLA-C* genes, which encode the human MHC-I complex. We extracted scripts from the Broad Institute’s Polysolver Docker container (https://software.broadinstitute.org/cancer/cga/polysolver_run). We verified that the majority of Polysolver’s HLA calls were consistent with that of xHLA [26] (Additional file 10: Figure S9) and therefore used all available Polysolver results. B2M mutations were taken directly from the TCGA MAF files. Patients with somatic B2M or HLA mutations were grouped for subsequent analysis and compared to patients that had neither. We found that only 13 patients had both B2M and HLA mutations (Additional file 9: Table S2).

### Microsatellite instability

Microsatellite instability scores for all TCGA patients were obtained from Kautto et al., 2017. Patient MANTIS scores from the paper were binarized to microsatellite instable (MSI-H) and stable (MSS) according to the recommended MANTIS score threshold of 0.4 [14].

### Determining expressed mutations

We used the bam-readcount tool (https://github.com/genome/bam-readcount) to determine how many RNAseq reads covered a mutated position. To count a mutation as being expressed, we used a read count threshold of 5.

### Determining regions with CNVs

Regions affected by copy number variants were determined from TCGA affymetrix SNP6 data by using 0.1 thresholds as the cutoff in either direction. Thus, any region that has a log2 fold change larger than 0.1 or smaller than − 0.1 is defined as a position with copy number variation [27]. For the Additional file 7: Figure S7 we excluded any mutations that occurred in regions with copy number variation.

### Mutation burden

Mutation counts were obtained from TCGA MAF files for all patients. To obtain nonsynonymous counts, we filtered out mutations outside of coding regions as well as silent mutations and tallied the remaining mutations for each patient. We retained only expressed mutations, and added a pseudocount of 1 for all patients, for all mutation burden analyses. For cancer-type-specific analysis, patients from TCGA tumor types COAD and READ were merged under the name CRC (colon and rectal cancer).

### Antigen affinity

We used the netMHCpan4.0 tool [15, 28] to obtain mutation affinity scores for all patient HLA alleles. To determine whether a mutation would be effectively bound as a neoantigen to the MHC-I complex, we binarized affinity scores: mutations with scores <= 2 we considered binding, and mutations with scores > 2 we considered non-binding [8, 15]. We then took the harmonic mean of the best ranking neoantigen to calculate the Patient Harmonic-mean Best Rank (PHBR) score [8]. To evaluate differences in fraction of binding neoantigens at various presentation score (PHBR-I score) cutoffs, we plotted the empirical cumulative distribution function (ECDF) using the median fraction of neoantigens generated from expressed mutations across patients. The Kolmogorov-Smirnov test was used to determine whether the distribution of neoantigen fractions was significantly different for each group (Fig. 4a). To determine if the number of neoantigens was significantly different between mutated and control patients at a particular PHBR-I score threshold, we calculated *p*-values using an unpaired Mann Whitney test for pan-cancer comparisons (Fig. 4b). To test the significance of the number of neoantigens between mutated and unmutated HLA alleles, we used a paired Wilcoxon test (4C, 4F). The Kolmogorov-Smirnov, Mann Whitney, and Wilcoxon tests implemented in the scipy.stats Python package were used for these analyses.

### Allelic fraction analysis

For Polysolver-determined HLA mutations, we obtained the tumor allelic fraction (“tumor_f”) from the Mutect output files generated by Polysolver. For all other mutations we calculated tumor allelic fraction from tumor alternate allele reads (“t_alt_count”) and tumor read depth (“t_depth”) from TCGA MAF files. B2M and HLA mutations were further annotated according to their percentile within the ranked list of mutations in the tumor where they were observed. To determine if the distributions of patients with B2M and HLA mutations were significantly different than patients without these mutations, we used an unpaired Mann Whitney statistical test from the scipy.stats Python package.

### Immune infiltration and cytotoxicity

Immune cell infiltration levels for CD8+ T cells and natural killer cells were obtained by running Cibersort with default parameters and without quantile normalization, on log2 TPM values obtained by reprocessing the TCGA RNAseq data through Sailfish V0.7.6 [29]. Cytotoxicity was estimated as described in [17], by summing the z-scored log2 TPM expression values of granzyme A (*GZMA*) and perforin (*PRF1*). For cancer-type-specific analysis, patients from TCGA tumor types COAD and READ were merged under the name CRC (colon and rectal cancer).

### Other statistical considerations

Where appropriate, p-values were adjusted for multiple comparisons using the Benjamini-hochberg method [30].

## Additional files

Additional file 1: **Figure S1.** MHC-I complex 3D structure. 3D crystal structure of MHC-I complex is displayed as B2M/HLA-A complex (PDB: 3bo8). Interface (blue and violet) and core (green and orange) regions of B2M and HLA-A proteins are highlighted, respectively. Transparent blue and violet regions correspond to the surface regions of B2M and HLA-A proteins, respectively. (PDF 1324 kb)

Additional file 2: **Figure S2.** B2M interface residue positions for HLA alleles. Residues on B2M that interact with HLA-A, HLA-B, HLA-C proteins are highlighted black. (PDF 381 kb)

Additional file 3: **Figure S3.** Increased mutation burden associated with mutations in HLA, related to Fig. 3. (A and B) Boxplots showing total number of nonsynonymous mutations for (A) MSI and MSS and (B) MSS only TCGA patients with or without HLA mutations for additional tissue types not shown in Figs. 3b, or d. Patients are divided by tumor type. Only the tumor types containing at least 5 mutated patients and that have not been reported in Fig. 3 are shown. *P*-values are adjusted for multiple comparisons using the Benjamini–Hochberg procedure. (PDF 435 kb)

Additional file 4: **Figure S4.** Tumor stage analysis for patients with B2M and HLA mutations. Percentage distribution of tumor stages for the patients with or without B2M and HLA mutations. (PDF 343 kb)

Additional file 5: **Figure S5.** Mutation burden in CCLE, related to Fig. 3. Boxplots showing the total number of nonsynonymous mutations for CCLE cell lines who acquired a B2M or HLA versus cell lines that did not acquire any B2M or HLA mutation. Sample sizes for each group are written under their name. (PDF 345 kb)

Additional file 6: **Figure S6.** Total number of binding neoantigens to patient HLA alleles, related to Fig. 4. (A) Distribution of median total counts of binding neoantigens at different PHBR-I score cutoffs for MSS patients. (B) Boxplots comparing the number of neoantigens in MSS patients with no B2M or HLA mutation (teal) versus MSS patients with a B2M mutation (yellow) or an HLA mutation (purple). A PHBR-I score cutoff of 2 was used to designate a binding neoantigen for this comparison. (PDF 419 kb)

Additional file 7: **Figure S7.** Allelic fraction percentile distribution for patients with B2M and HLA mutations accounting for aneuploidy, related to Fig. 4. Allelic fraction percentile distribution for expressed mutations in MSS patients with B2M and HLA mutations, excluding all mutations occurring in regions affected by CNVs. Patients that have both B2M and HLA mutations are excluded. (PDF 330 kb)

Additional file 8: **Figure S8.** NK, CD8+ T-cell and cytotoxicity levels of patients with mutations in HLA, related to Fig. 5. (A-B-C) Boxplots comparing MSS TCGA patients with or without HLA mutations for additional tissue types not shown in Fig. 5(B-D-F), in terms of their (A) CD8+ T-cell levels, (B) NK cell levels, and (C) cytotoxicity scores. Patients are divided by tumor type and only the tumor types with at least 5 mutated patients and that have not been reported in Fig. 5 are shown. P-values are adjusted for multiple comparisons using the Benjamini–Hochberg procedure. (PDF 428 kb)

Additional file 9: **Table S1.** Summary of data availability for specific analyses. List of TCGA patients analyzed in the manuscript, annotated according to data availability for inclusion in specific analyses. **Table S2.** List of patients with B2M or HLA mutations. Duplicate patient mutation rows indicate that both HLA alleles are mutated. (XLSX 331 kb)

Additional file 10: **Figure S9.** HLA allele call comparison between Polysolver and xHLA. Barplot showing matched Polysolver HLA calls and calls with major and minor subtype differences. (PDF 337 kb)

## Data Availability

All available data were downloaded from The Cancer Genome Atlas (TCGA) database via their Genomic Data Commons (GDC) client. No novel data were generated.

## Abbreviations

B2M: Beta-2-microglobulin
CCLE: Cancer Cell Line Encyclopedia
CRC: Colon and rectal cancer
CTLs: Cytotoxic T cells
ECDF: Empirical cumulative distribution function
GDC: Genomic Data Commons
LOH: Loss of heterozygosity
MHC-I: Major histocompatibility complex class I
MSI: Microsatellite instability
MSI-H: Microsatellite instable
MSS: Microsatellite stable
NK: Natural killer
PDB: Protein Data Bank
PHBR: Patient Harmonic-mean Best Rank
RSA: Relative solvent accessible surface area
TCGA: The Cancer Genome Atlas
TCR: T cell receptor
VAF: Variant allele fraction
WXS: Whole exome sequencing
