# Supplementary figures and images for: Elevated neoantigen levels in tumors with somatic mutations in the HLA-A, HLA-B, HLA-C and B2M genes
Source: BMC Med Genomics. 2019 Jul 25;12(Suppl 6):107. doi: 10.1186/s12920-019-0544-1 (PMC6657029; doi:10.1186/s12920-019-0544-1)

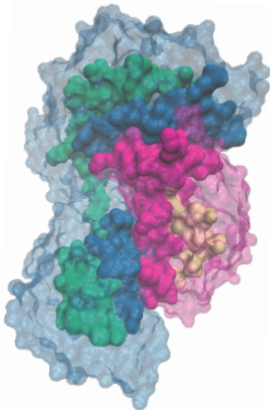

B2M

Surface  
Interface  
Core

HLA

Surface  
Interface  
Core

Supplement: Supplementary file 1 — Figure S1. MHC-I complex 3D structure. 3D crystal structure of MHC-I complex is displayed as B2M/HLA-A complex (PDB: 3bo8). Interface (blue and violet) and core (green and orange) regions of B2M and HLA-A proteins are highlighted, respectively. Transparent blue and violet regions correspond to the surface regions of B2M and HLA-A proteins, respectively. (PDF 1324 kb) [file 12920_2019_544_MOESM1_ESM.pdf]

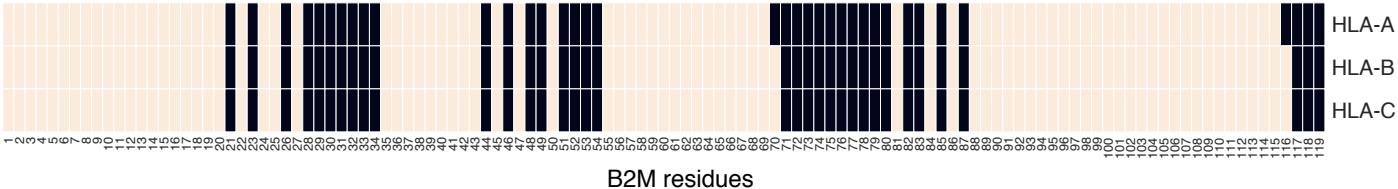

Supplement: Supplementary file 2 — Figure S2. B2M interface residue positions for HLA alleles. Residues on B2M that interact with HLA-A, HLA-B, HLA-C proteins are highlighted black. (PDF 381 kb) [file 12920_2019_544_MOESM2_ESM.pdf]

**A**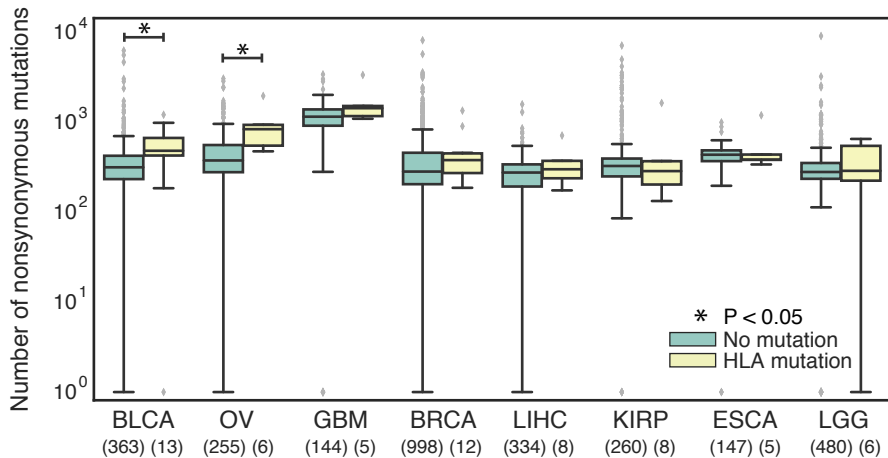**B**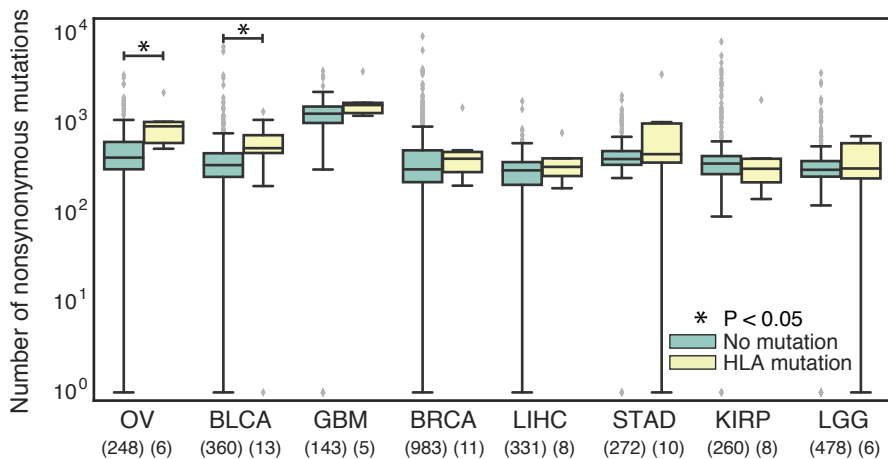

Supplement: Supplementary file 3 — Figure S3. Increased mutation burden associated with mutations in HLA, related to Fig. 3. (A and B) Boxplots showing total number of nonsynonymous mutations for (A) MSI and MSS and (B) MSS only TCGA patients with or without HLA mutations for additional tissue types not shown in Figs. 3b, or d. Patients are divided by tumor type. Only the tumor types containing at least 5 mutated patients and that have not been reported in Fig. 3 are shown. P-values are adjusted for multiple comparisons using the Benjamini–Hochberg procedure. (PDF 435 kb) [file 12920_2019_544_MOESM3_ESM.pdf]

Percentage distribution  
of tumor stages (%)

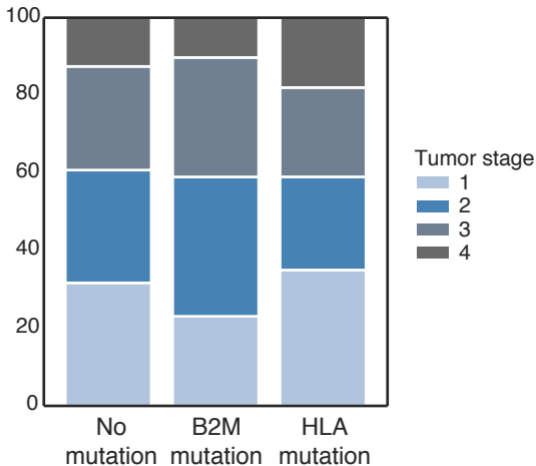

Supplement: Supplementary file 4 — Figure S4. Tumor stage analysis for patients with B2M and HLA mutations. Percentage distribution of tumor stages for the patients with or without B2M and HLA mutations. (PDF 343 kb) [file 12920_2019_544_MOESM4_ESM.pdf]

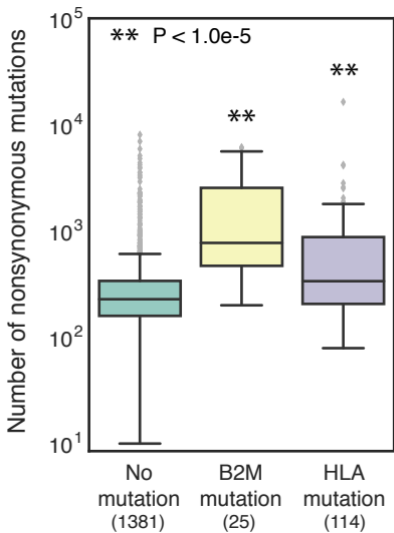

Supplement: Supplementary file 5 — Figure S5. Mutation burden in CCLE, related to Fig. 3. Boxplots showing the total number of nonsynonymous mutations for CCLE cell lines who acquired a B2M or HLA versus cell lines that did not acquire any B2M or HLA mutation. Sample sizes for each group are written under their name. (PDF 345 kb) [file 12920_2019_544_MOESM5_ESM.pdf]

**A**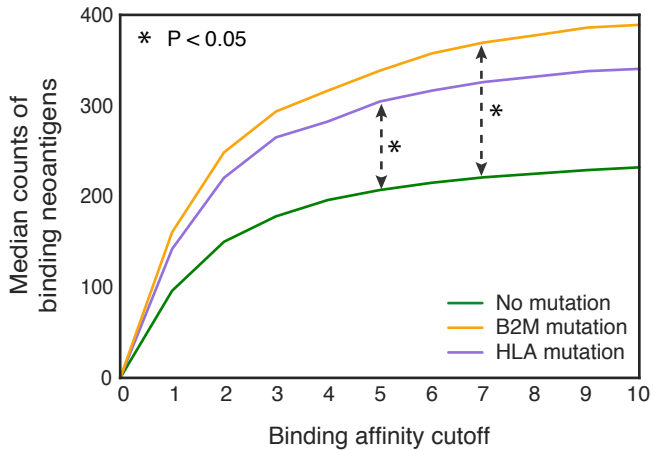**B**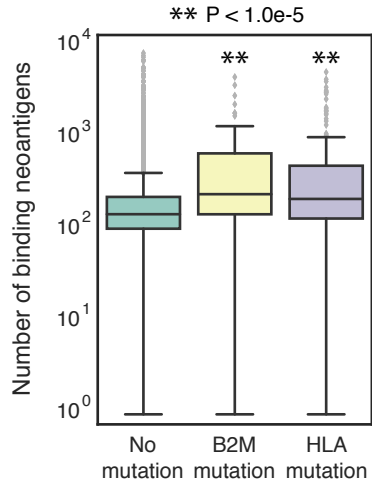

Supplement: Supplementary file 6 — Figure S6. Total number of binding neoantigens to patient HLA alleles, related to Fig. 4. (A) Distribution of median total counts of binding neoantigens at different PHBR-I score cutoffs for MSS patients. (B) Boxplots comparing the number of neoantigens in MSS patients with no B2M or HLA mutation (teal) versus MSS patients with a B2M mutation (yellow) or an HLA mutation (purple). A PHBR-I score cutoff of 2 was used to designate a binding neoantigen for this comparison. (PDF 419 kb) [file 12920_2019_544_MOESM6_ESM.pdf]

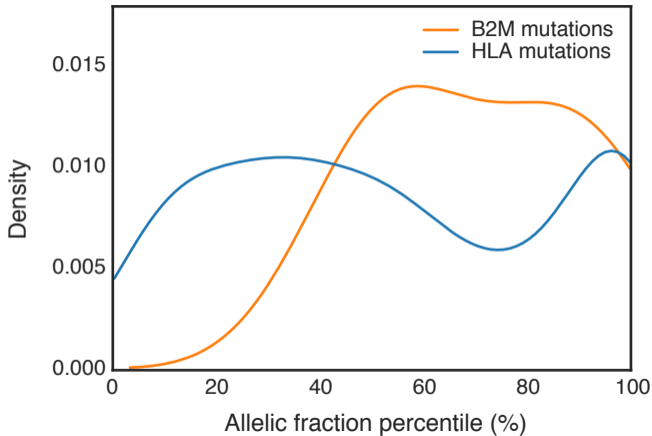

Supplement: Supplementary file 7 — Figure S7. Allelic fraction percentile distribution for patients with B2M and HLA mutations accounting for aneuploidy, related to Fig. 4. Allelic fraction percentile distribution for expressed mutations in MSS patients with B2M and HLA mutations, excluding all mutations occurring in regions affected by CNVs. Patients that have both B2M and HLA mutations are excluded. (PDF 330 kb) [file 12920_2019_544_MOESM7_ESM.pdf]

**A**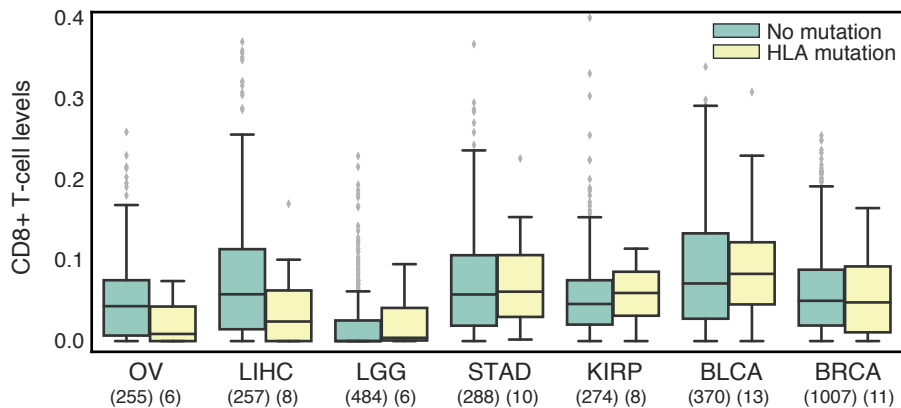**B**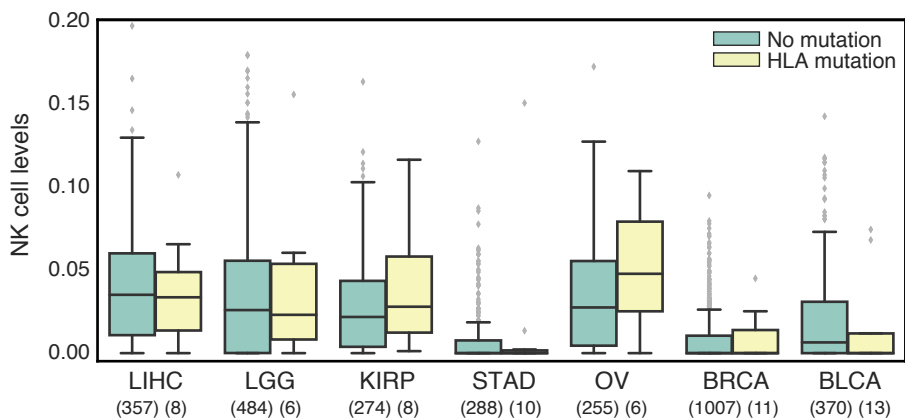**C**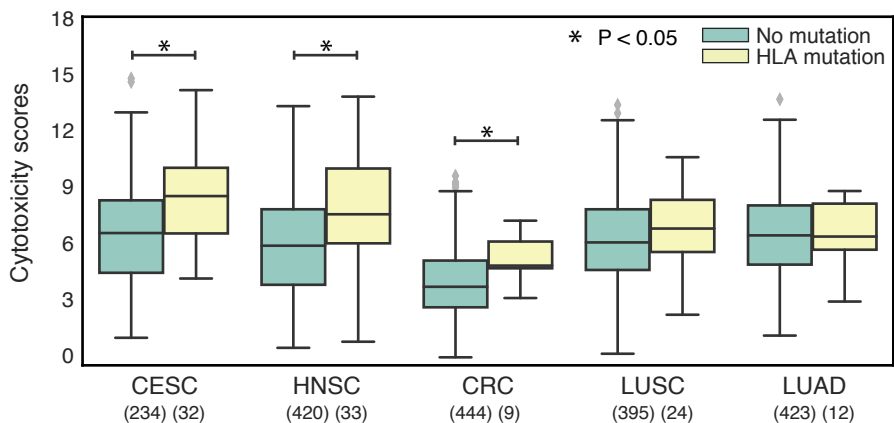

Supplement: Supplementary file 8 — Figure S8. NK, CD8+ T-cell and cytotoxicity levels of patients with mutations in HLA, related to Fig. 5. (A-B-C) Boxplots comparing MSS TCGA patients with or without HLA mutations for additional tissue types not shown in Fig. 5(B-D-F), in terms of their (A) CD8+ T-cell levels, (B) NK cell levels, and (C) cytotoxicity scores. Patients are divided by tumor type and only the tumor types with at least 5 mutated patients and that have not been reported in Fig. 5 are shown. P-values are adjusted for multiple comparisons using the Benjamini–Hochberg procedure. (PDF 428 kb) [file 12920_2019_544_MOESM8_ESM.pdf]

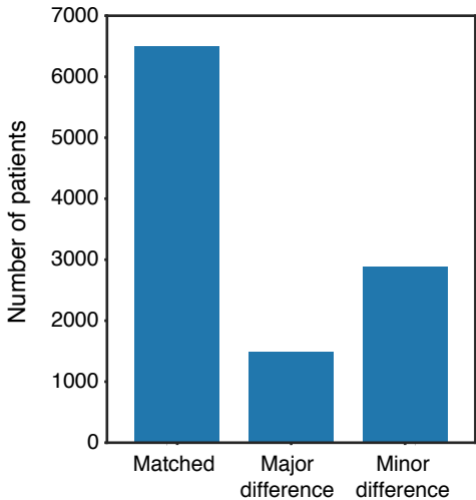

Supplement: Supplementary file 10 — Figure S9. HLA allele call comparison between Polysolver and xHLA. Barplot showing matched Polysolver HLA calls and calls with major and minor subtype differences. (PDF 337 kb) [file 12920_2019_544_MOESM10_ESM.pdf]
